# Preclinical delayed toxicity studies of BCMA CAR T-cell injection in B-NDG mice with multiple myeloma

**DOI:** 10.3389/fimmu.2024.1435934

**Published:** 2024-11-13

**Authors:** Jianmin Guo, Qiqi Wu, Hongjian Li, Chun Liang, Jinlong Dai, Shuren Zhang, Cailing Dai, Jishuai Zhang, Yuying Wen, Wei Yang

**Affiliations:** ^1^ Division of Life Science and State Key Lab of Molecular Neuroscience, Hong Kong University of Science and Technology, Hong Kong, Hong Kong SAR, China; ^2^ Guangzhou Bay Area Institute of Biomedicine, Guangdong Lewwin Pharmaceutical Research Institute Co.,Ltd., Guangdong Provincial Key Laboratory of Drug Non-Clinical Evaluation and Research, Guangdong Engineering Research Center for Innovative Drug Evaluation and Research, Guangzhou, China; ^3^ Guangdong Engineering Research Center for Cellular and Genetic Therapy Innovative Drugs, Shenzhen, China; ^4^ College of Pharmacy, Guilin Medical University, Guilin, China; ^5^ Shenzhen Pregene Biopharma Company Ltd., Research and Development (R&D) Department, Shenzhen, China

**Keywords:** multiple myeloma, chimeric antigen receptor T cells, immunotherapy, B-cell maturation antigen, cytokines, delayed toxicity

## Abstract

**Purpose:**

Based on the efficacy data from the previous study of B-cell maturation antigen (BCMA) chimeric antigen receptor (CAR) T-cell injection, we further examined the delayed toxicity for 8 weeks after a single dose of BCMA CAR T-cell injection to observe possible toxic reactions.

**Methods:**

B-NDG mice transplanted with multiple myeloma (MM) cells were given a single dose of BCMA CAR T-cell injection at two dosages or human normal T cells and then subjected to examinations including clinical signs, weight and food intake measurements, haematology, blood biochemical analysis, cytokine assay, T-lymphocyte subpopulation quantification and histopathology on days 28 and 56 after dosing. In addition, quantitative polymerase chain reaction (qPCR) was used to quantify DNA fragments in different tissues to assess the tissue distribution of CAR and provide a basis for its preclinical safety evaluation and clinical dosing.

**Results:**

In the delayed toxicity study, no mortality or significant toxic effects such as reductions in food intake, body weight, relevant biochemical parameters and target organ weights were observed in the BCMA CAR T-cell-treated groups. Compared to the model group, restorative changes in clinical signs and clinicopathology indicating therapeutic effects were seen in the BCMA CAR T-cell-treated groups. Human-derived cytokines interleukin-2 (IL-2), IL-4, IL-6, IL-12, IL-10, tumor necrosis factor α (TNF-α), and interferon-γ (IFN-γ) could be detected in all cancer cell–bearing mice by cytokine level measurement. IFN-γ levels showed a geometric increase due to the graft versus host disease (GVHD) response induced in the mice, while the levels of the other cytokines did not show significant changes. Histopathological examination indicated that the BCMA CAR T-cell treatment groups showed mixed cellular infiltration of human-derived T cells, cancer cells, and inflammatory cells in several target organs including the liver, spleen, lung, and kidney, and some of them showed mild tissue damage, but the number of the animals and the severity of damage were significantly less than those of the T-cell control group as well as the model group. The results of the tissue distribution study showed that BCMA CAR T cells were mainly concentrated in the kidney, lung, bone marrow and the related immune organs/tissues, and the distribution of BCMA CAR T cells was highly consistent with that of MM cells, suggesting that BCMA CAR T cells could follow the cancer cells during metastatic targeting of the tissues.

**Conclusions:**

The present study demonstrated a low toxicity of BCMA CAR T-cell injection, with manageable side effects and good anticancer activity and without observable adverse effects. This study provides data to support future clinical studies of BCMA CAR T-cell injection for MM.

## Introduction

Multiple myeloma (MM) is the second most common haematological malignancy worldwide, accounting for approximately 1.8% of all cancers (1–5), and is characterised by the presence of uncontrolled growth of clonal plasma cells in the bone marrow, leading to destruction of bone mass, kidney damage, anaemia, and hypercalcaemia in patients. The disease is common in people aged 65–74 years, with a median age of 69 years, and is more common in men than women (6). According to epidemiological investigations, the occurrence of MM may be related to obesity, exposure to pesticides or organic solvents, ionising radiation, heredity, genetic mutations, viral infections, and environmental changes (7–11).

Significant advances in the treatment of MM over the last decades have led to increasing survival rates and greatly improved patient prognosis, with overall survival rates increasing from 32% in 1996 to 54% in 2020 (12, 13). A growing number of drugs are now available for the treatment of MM, including proteasome inhibitors (carfilzomib and isazomib), antibody-coupled drugs (daratumumab and elotuzumab), immunomodulatory drugs (thalidomide and lenalidomide), histone deacetylase inhibitors (panobinostat), completely novel drugs such as nuclear export protein inhibitor-1 (XPO-1, Swlinexor), and cellular immunotherapy (Abecma and Carvykti), all of which have shown significant anticancer efficacy in the clinical management of MM (14–16). Despite the continuous advancement of the new therapeutic agents, which can significantly prolong survival in MM during initial treatment, residual disease persists and equilibrates with the host immunity in the later stages of treatment due to the heterogeneous nature of MM. Some patients often end up relapsing due to drug resistance, while others experience significant treatment-related toxicities resulting in a life-threatening cytokine storm, which is the most significant challenge encountered in the treatment of MM. Therefore, the search for more effective, safer, more specific, and longer lasting MM therapy is an urgent need.

In the recent years, cellular immunotherapy has been considered an effective modality for the treatment of B-cell malignancies, among which chimeric antigen receptor (CAR) T cells, which has made great progress in many clinical trials on MM, is a very promising therapy in the treatment of relapsed/refractory haematological malignancies that have failed to respond to all other drugs. CAR-T has been successfully used to treat other haematological diseases, malignant lymphoma, acute lymphoblastic leukaemia (ALL), and diffuse large B-cell lymphoma (DLBCL), and has been approved by the U.S. Food and Drug Administration (FDA) and European Medicines Agency (EMA) (17). CAR T-cell therapy is a treatment in which peripheral blood is collected from patients, and the isolated T cells are genetically modified and expanded *in vitro* and transfused back into the patient to direct T-cell proliferation and recognition of target antigens on the surface of cancer cells, which in turn causes killing of cancer cells (18, 19).

As a very important target of MM, B-cell maturation antigen (BCMA/CD269) is a member of the TNF receptor super family and is present on the surface of myeloma, plasma cells, and terminally differentiated plasma cells but not on the surface of other normal tissues or vital organs (20–22). It has been shown that among several validated MM targets such as SLAMF and CD38, only BCMA expression is upregulated in MM progression (23–25), a feature that satisfies a key criterion in CAR T-cell design, suggesting that BCMA is one of the most promising targets for the treatment of MM with CAR-T. Because of its low potential systemic and local side effects, BCMA is more often targeted in CAR T-cell therapy compared to other targets. Clinical efficacy and safety data for the treatment of MM/RR MM (relapsed/refractory MM) with BCMA CAR T cells are good, with high response rates and low incidence of adverse events, but a series of lethal toxic reactions (e.g., cytokine release syndrome (CRS), neurotoxicity, graft versus host disease, and chronic renal failure) in some patients due to the escape of MM cells caused by reduced BCMA expression or loss of shedding are still inevitable (26–28). Therefore, the development of BCMA CAR-T cell therapy should not only maximize the durability and target specificity but also focus on the safety.

In a previous study, our group conducted a preclinical study on the efficacy and acute treatment phase toxicity of BCMA CAR T-cell injection in a B-NDG immune-deficient mouse model of MM transplantation, which showed no significant toxic reactions during the acute treatment (unpublished data, by J. S. Zhang, H. J. Li et al.). The results suggest that BCMA CAR T-cell injection may be a potentially better form of secondary immunotherapy for patients with MM. In this study, we further investigated the delayed toxicities of a single administration of BCMA CAR T-cell injection on days 28 and 56 in the B-NDG mouse model with MM to examine the possible toxic responses during progression-free survival and relapse after treatment.

## Materials and methods

### Target cell lines

The MM cell line (MM.1S) was obtained from ATCC, and the luciferase (Fluc) vector was designed and generated by Shenzhen Pregene Biopharmaceutical Co. Cells were cultured in RPMI-1640 medium containing 10% fetal bovine serum (FBS) and 1% penicillin-streptomycin solution in an incubator at 37°C and 5% CO_2_. The cells were STR-identified prior to the experiments and were consistent with the cell line’s characteristics. BCMA CAR T cells and normal human T cells were provided by Pregene (Shenzhen) Biotechnology Co.

### Screening and humanization of anti-BCMA VHH

The BCMA-specific VHH antibody was screened from a phage display alpaca library with three rounds of panning. The gene sequences were subsequently transferred into the prokaryotic expression vector. ELISA and flow cytometry were used to screen candidate sequences, and Octet RED system (ForteBio, Fremont, CA, USA) was used to measure the affinity. Finally, one candidate sequence was selected for incorporation into the CAR vector. The selected VHH sequence was humanized by replacing several amino acids with homologous human heavy chain in the framework region.

### Preparation of CAR T cells

T cells were isolated from the leukapheresis products of the healthy volunteers by using CD3 microbeads (Miltenyi Biotec B.V. & Co. KG, Germany) and subsequently stimulated with pre-coated Retro Nectin (20 μg/mL, GMP grade, Takara Bio Inc, Shiga, Japan) and anti-CD3 antibody (5 μg/mL, GMP grade, Takara Bio Inc, Shiga, Japan) on a six-well plate (Corning Inc. - Life Sciences, Kennebunk, ME, USA) in X-VIVO 15 medium (Lonza, Basel, Switzerland) supplemented with IL-2 (1000 IU/mL, Shandong Quan gang Pharmaceutical Co. Ltd., Shandong, China) and 3% autologous serum. The next day, T cells were transduced a lentiviral vector containing a humanized anti-BCMA VHH, CD8α extracellular and transmembrane domain, 4-1BB cytoplasmic domain, and CD3ζ cytoplasmic domain. Based on the cell density adjustment, we expanded the CAR T cells by supplementing the X-VIVO 15 medium with IL-2 (1000 IU/mL) and 1% autologous serum. On day 12, CAR-T was harvested after centrifugation.

**Figure 1:**
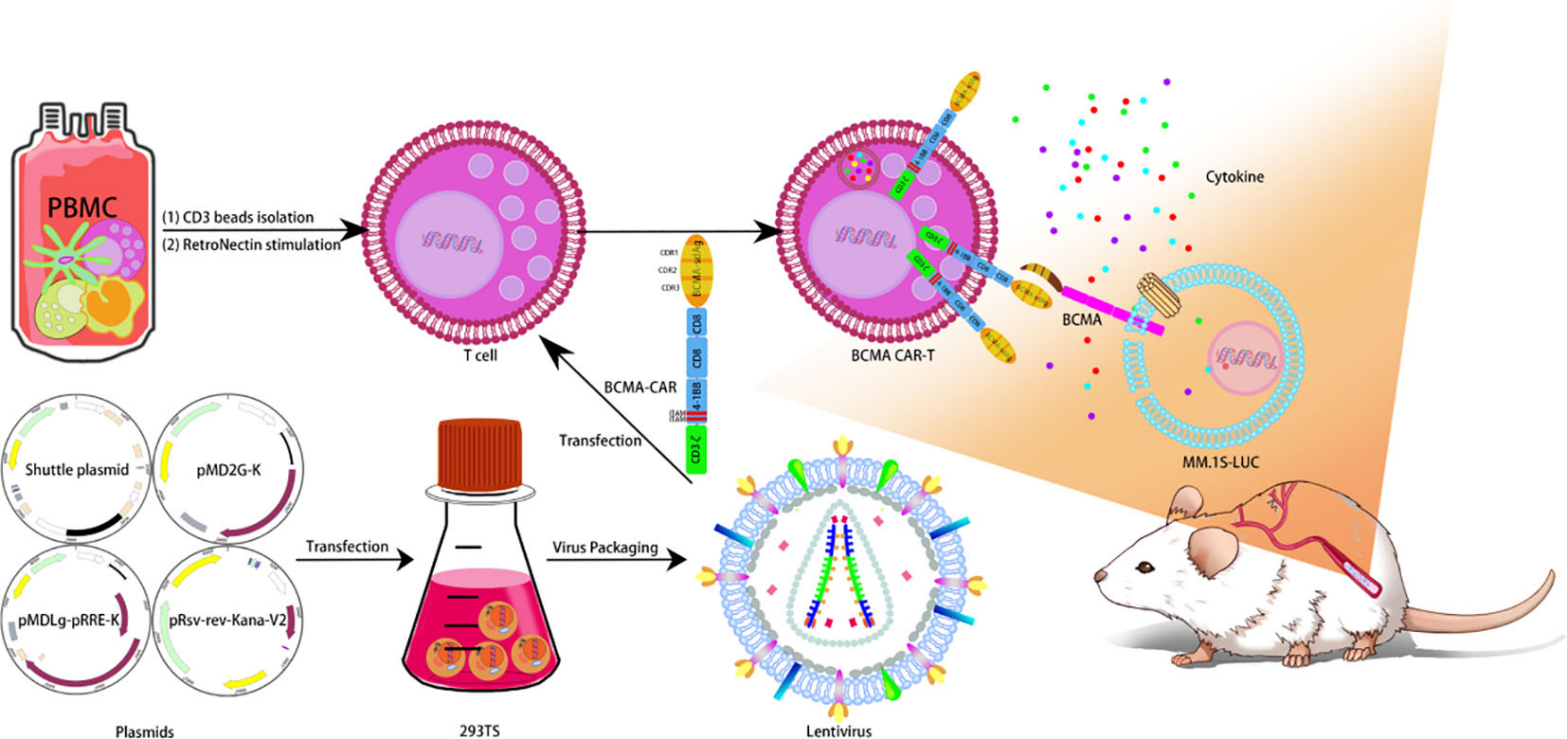
Design of BCMA CAR T cells. Lentivirus was produced by suspension 293TS cells, which transfected with a four-plasmid system. For CAR T-cell manufacture, T cells were isolated from PBMC by CD3 microbeads as described in method, and then transduced with lentivirus. The CAR-transduced T cell will express designed anti-BCMA CAR components on the cell membrane. For mouse toxicity studies, B-NDG mice were transplanted with MM.1S-LUC cells and given a single dose of BCMA CAR T-cell injection.

Lentivirus was produced by suspension 293TS cells which transfected with a four-plasmid system. For CAR T-cells manufacture, T cells were isolated from PBMC by CD3 microbeads as described in method, and then transduced with lentivirus. The CAR-transduced T cell will express designed anti-BCMA CAR components on the cell membrane. For mouse toxicity studies, B-NDG mice were transplanted with MM.1S-LUC cells and given a single dose of BCMA CAR T-cell injection (**Figure 1**).

### Animals

The 4-week-old B-NDG–humanized mice (16.2–28.1 g) used in this study were sourced from Biosetu Jiangsu Genetic Biotechnology Co. Animal certificate number: SYXK (Guangdong) 2015-0146. Mice were housed in an specific pathogen-free (SPF)-rated facility according to the Animal Care Facility Guidelines. The experiments were reviewed and approved by the Institution’s Animal Care and Use Committee, IACUC, in strict accordance with AAALAC requirements and were conducted under the relevant FDAGLP and NMPA GLP regulations. All environmental specifications met the requirements of the institution’s environmental specifications. All animal experiments were conducted in accordance with guidelines for animal welfare, and the animals were used in cancer research with humane care and treatment. Individually ventilated cages were used for the mice. The animal room has the ambient temperature of 20.0°C–24.3°C, relative humidity 40%–70.0%, air changes ≥ 15 times/h, and 12 h of alternating light and dark.

B-NDG immuno-deficient mice are internationally recognized for their high degree of immunodeficiency and suitability for transplantation of human-derived cells or tissues due to their lack of immune cells such as mature T cells, B cells, and NK cells compared to other immune-deficient mice, especially as the knockout of the gamma chain of the IL-2 receptor further reduces the immune function in mice. In the current delayed toxicity study, B-NDG mice were transplanted with MM.1S cells expressing Luciferase protein and randomly grouped according to the intensity of fluorescence to facilitate subsequent experiments.

### Delayed toxicity studies

Some 250 B-NDG mice (125 males and 125 females) were used for the experiments. Fifty mice (25 males and 25 females) were randomly chosen for the non-treated negative control group, and 200 mice were injected with 10 ml/kg body weight of MM.1S-Luc cells at 1 × 10^7^ cells/mL via the tail vein. After confirmation of MM cell transplantation by bioluminescence imaging (over 70% of the mice were positive, which implied a near 100% success rate of transplantation, as the detection limit was 1 × 10^6^ cells), the animals were randomly divided into four groups of 50 (25 males and 25 females) each according to their body weight and sex. There were five groups of animals in this study with 50 mice in each group: negative control group (without cancer cells), model control group, normal T-cell group, low-dose BCMA CAR-T group, and high-dose BCMA CAR-T group. Ten animals per group were used for blood collection only, and the remaining 40 mice per group were used for the main tests.

Each animal in the three groups with MM cells was injected with a low (5 × 10^7^ cells/kg) or a high (5 × 10^8^ cells/kg) dose of BCMA CAR T cells or human T cells (5 × 10^8^ cells/kg) at 10 mL/kg, and the mice were closely observed clinically for 2–4 h after administration and the next day. Blood collection from the mice was performed on days 28 and 56 after administration. To assess toxicity, whole blood and serum were used for haematology and blood biochemical analysis, including the following indices: red blood cell count (RBC), hemoglobin concentration (HGB), red blood cell pressure volume (HCT), mean red blood cell volume (MCV), mean red blood cell hemoglobin content (MCH), mean red blood cell hemoglobin concentration (MCHC), red blood cell volume distribution width SD (RDW-SD), reticulocyte absolute value (RET#), immature reticulocyte ratio (IRF), medium fluorescent reticulocyte ratio (MFR), high fluorescent reticulocyte ratio (HFR), white blood cell count (WBC), platelet system and coagulation index platelet count (PLT&O), alanine aminotransferase (ALT), aspartate aminotransferase (AST), albumin (ALB), urea nitrogen (BUN), creatine phosphokinase (CK), glutamate dehydrogenase (GLDH), Ca^2+^, K^+^, and Cl^−^. The animals in the main tests were subjected to systematic dissection, and major organs (e.g., whole blood, liver, spleen, lungs, kidneys, bone marrow, brain) were taken for histopathological studies. Body weight and food intake of all animals were also monitored throughout the study and prior to their execution. The animals were weighed on days 2, 3, 5, 7, 11, 14, and weekly, and the amounts of food added to each cage were recorded throughout the experimental period. The feed given to each cage was recorded after 24 h and the corresponding residual feed per cage was used to calculate the daily feed intake.

### Immunotoxicity studies

Twenty mice (10 males and 10 females) per group described in the “Delayed toxicity studies” were dissected on days 28 and 56, and the blood and organs were collected. Before blood collection, the animals were fasted for approximately 14–16 h and anesthetized with isoflurane. Whole blood was taken for T-lymphocyte population analysis. Cytokine assays were performed on days 1, 3, 14, 28, and 56 after administration for blood collection. Bone marrow cells were collected from the left femur by repeatedly flushing of the marrow cavity using inactivated FBS, placed in centrifuge tubes, and then assayed for bone marrow cell sorting using the XN-1000V fully automated blood and body fluid hematology analyser (Sysmex). Histopathological examination and weighing of the relevant immune tissues and organs (mesenteric lymph nodes, inguinal lymph nodes, spleen, thymus, etc.) were performed. Both the T-lymphocyte subpopulation assay and cytokine assay were performed using a CytoFLEX flow cytometer. In the T-lymphocyte subpopulation assay, red blood cell lysis buffer was used to lyse the blood samples for measuring CD3^+^, CD4^+^, and CD8^+^ cells, and CytoFLEX analysis software was used to analyse the data. Cytokines including IFN-γ, IL-2, IL-4, IL-6, IL-12, IL-10, and TNF-α were measured using a human cytokine kit (Dakowei Biotechnology Co., Ltd.).

### Tissue distribution studies

Tissue distribution studies were carried out using the animals and samples from the “delayed toxicity studies” and “immunotoxicity studies.” Blood, major tissues and organs (including the spleen, lung, liver, brain, kidney, uterus, ovary, testis, epididymis, etc.) were collected from the mice in the low and high dose BCMA CAR T-cell injection groups on days 28 and 56 post-dosing and used for DNA isolation according to the blood/cell/tissue genomic DNA extraction kit (TIANGEN) instructions. Isolated DNA fragments were analysed by real-time quantitative polymerase chain reaction (RT-qPCR) to detect the copy number of the CAR gene in different tissues and to observe the distribution of CAR^+^ cells in each tissue. The upstream and downstream primer sequences for BCMA CAR-T were (GGCACTGACAATTCCGTGGT, AGGGACGTAGCAGAAGGACG), and the probe sequence was (ACGTCCTTTCCATGGCTGCTCGC). The primers and probes were designed and synthesized by Guangzhou Aiki Biotechnology Co.

### Statistical analysis

All data were entered into EXCEL for statistical purposes, and means ± standard deviations were calculated for haematology, serum biochemistry, body weight, food intake, leucocyte sorting counts, T-cell sorting counts, and relevant human cytokine levels in different genders and groups. F-test analysis was performed prior to group comparisons for each experimental group. When the variance between groups was equal, the Student t-test was used for statistical analysis, and when the variance between groups was not equal, the corrected Student t-test was used for statistical analysis. Pathological examination abnormalities were compared between the groups according to their incidence and degree.

## Results

### No mortality or significant toxic effects due to CAR T cells in CAR T-cell-treated B-NDG mice

**Figure 2:**
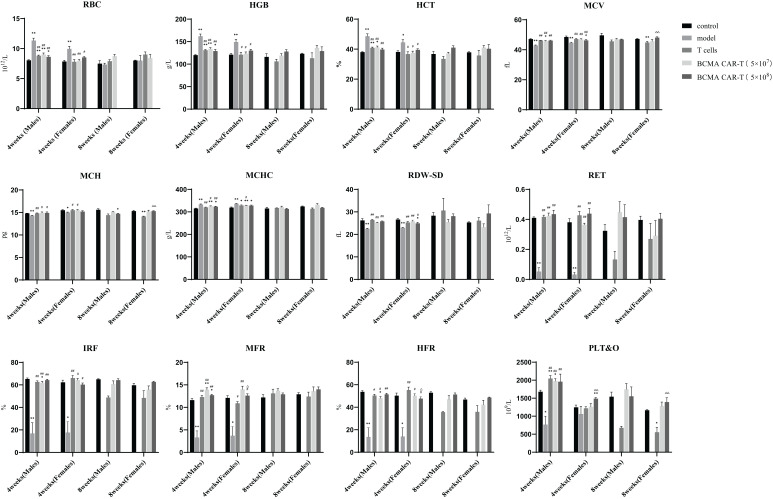
Summary of hematological indices of BCMA CAR T-cell injection on multiple myeloma xenograft mouse models (males and females, *n* = 5 for each group and each time point, x ± S). This figure shows the hematological indicators of male and female mice at fourth week and eighth week, divided into five groups, control, model group, T-cell group, BCMA CAR-T low-dose and high-dose group. All mice in the model group were euthanized before week 8, so no data was available. Compared with control group, *P < 0.05, **P < 0.01, compared with model group, ^#^ *P* < 0.05, ^##^ *P* < 0.01, compared with T cells control group, ^△^ *P* < 0.05, ^△△^ *P* < 0.01. Fourth week and eighth week indicate that these data were obtained on day 28 and day 56, respectively, after dosing.

**Figure 3:**
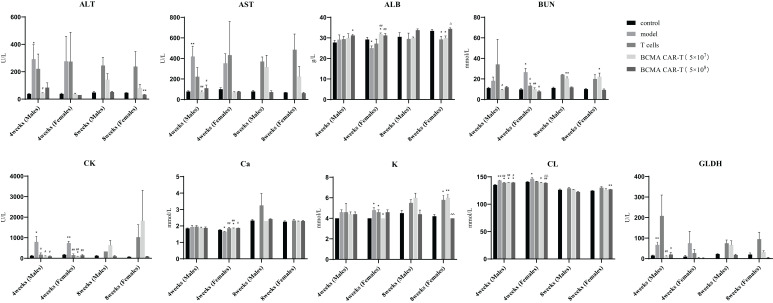
Summary of blood biochemical parameters of BCMA CAR T-cell injection on multiple myeloma xenograft mouse models (males and females, *n* = 5 for each group and each time point, x ± S). This figure shows the blood biochemical parameters of male and female mice at fourth week and eighth week, divided into five groups, control, model group, T-cell group, BCMA CAR-T low-dose and high-dose group. All mice in the model group were euthanized before week 8, so no data were available. Compared with control group, *P < 0.05, **P < 0.01; compared with the model group, ^#^ *P* < 0.05, ^##^ *P* < 0.01 compared T-cell group, ^△^ *P* < 0.05, ^△△^ *P* < 0.01. fourth week and eighth week indicate that these data were obtained after days 28 and 56.

During the whole experimental period (see **Figure 1** for the experimental procedures), animals in the model group began to show signs of cancer wastage such as vertical hair, wasting, arching of the back, and slow movement on days 19–21. On day 24, the mice began to die, and on day 27, a significant weight loss (> 20%) began to occur. Animals with significant weight loss were euthanized on day 27, blood was collected, and organ samples were obtained by autopsy. In the model control group, relevant haematological indicators such as MCV, MCH, IRF, MFR, HFR decreased, other indicators such as RBC, WBC, AST, ALT, CK increased, and platelet system and coagulation function indicators such as platelet count (PLT&O, RDW-SD) decreased (**Figures 2** and **3**).

**Figure 4:**
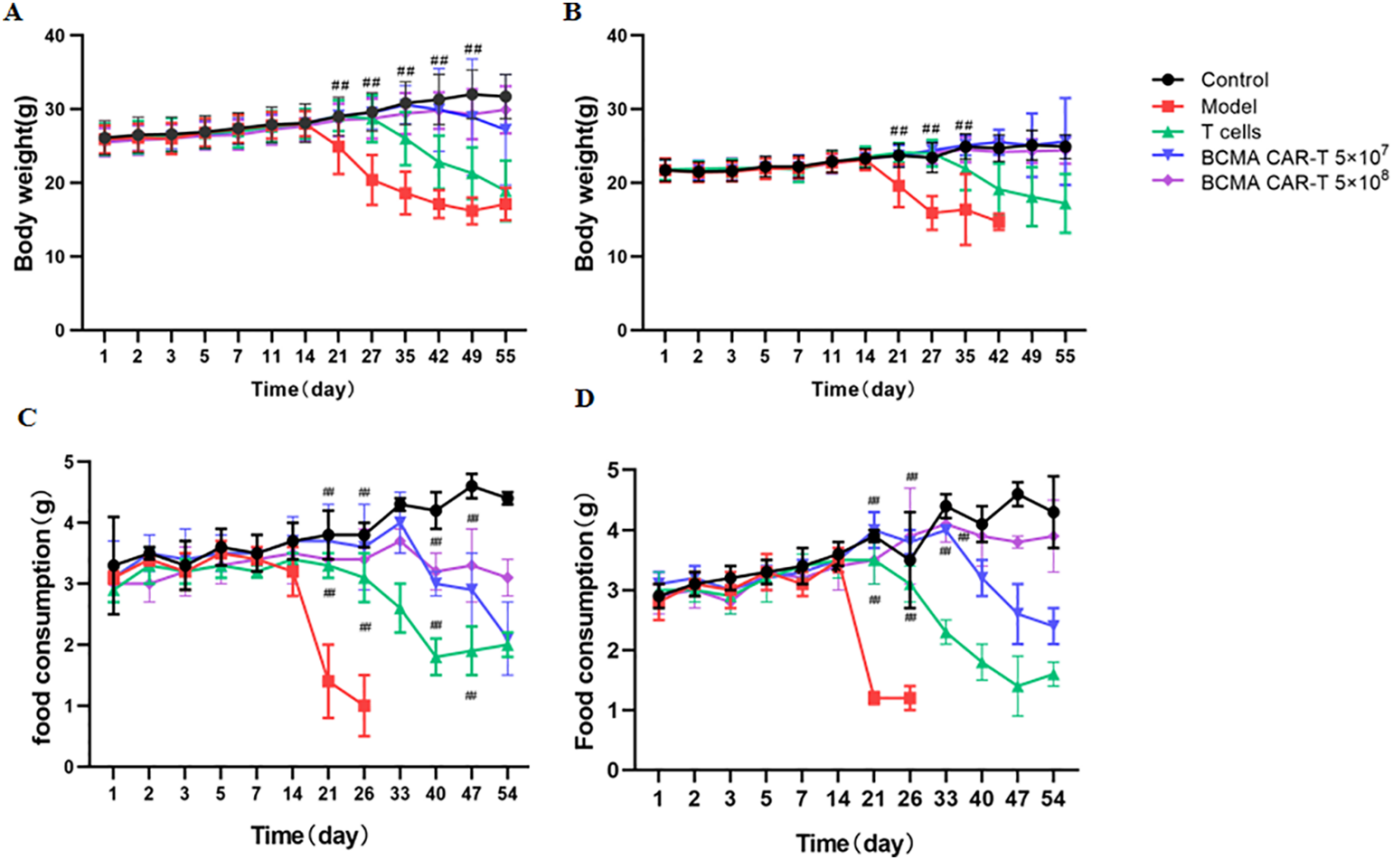
Body weight changes in B-NDG mice on day 56 after administration (*n* = 20 for each group). Changes in body weight of mice **(A, B)**, and food consumption **(C, D)** in the groups were determined. Mice administered with BCMA CAR T-cell injection lost weight more slowly compared to the model group (^##^ *P* < 0.01). ^##^ *P* < 0.01, comparing to the model group. In addition, the number of surviving mice in the model group on day 33 was only one to two per cage, and therefore the food intake data were not available.

The BCMA CAR T-cell low-dose low dose group animals began to show cancer wasting signs such as vertical hair, lethargy, arching of the back and slow movement on day 27 after dosing, and similar signs were observed in the BCMA CAR T-cell high-dose high dose group two weeks later (starting from day 42).The body weights of the CAR T-cell-treated group animals were significantly higher than those of the model control and normal T-cell control group and were not significantly different from the negative control group (**Figure 4**). Food consumption in the two CAR T-cell-treated groups was significantly higher than that of the model control groups on day 21 (**Figure 4**). These data indicate that cancer attrition occurred in the CAR T-cell-treated groups significantly later than the model group in a dose-dependent manner. This suggests that BCMA CAR T cells do not have toxicologically significant effects on animals while having anticancer effects.

Haematological indicators were analyzed on days 28 and 56 after administration (**Figure 2**). On day 28, the erythrocyte system indicators RBC, HGB, HCT, and MCHC were lower in the BCMA CAR T-cell groups than in the model control group (*P* < 0.05 or *P* < 0.01), and no significant differences in the high dose group were observed comparing to the negative control. Some differences in these indicators between the BCMA CAR T-cell groups and the model control group may be due to the pharmacological anticancer effects of the BCMA CAR T cells in the mice, instead of toxicological significance. The levels of WBC, NEUT#, LYMPH#, MONO#, and MONO% in the leukocyte system were higher than those in the negative control group. In the platelet system, males and females had higher PLT&O levels than the negative control (*P* < 0.01). Female animals had higher PLT&O levels and lower MCV levels than negative controls, both hematological indicators being possibly associated with cancer cell infiltration and proliferation in the animals. On day 56 after administration, males had higher MONO% levels than negative controls, lower MCH and NEUT% levels than negative controls, and higher PLT&O levels than negative controls in females. Most of the other haematological indices were not significantly different from the negative control group, and the relevant indices mostly turned to normal, which was likely due to the changes in the cancer cell load. These results indicate that restorative changes in clinical signs and clinic pathology due to the therapeutic effects of the BCMA CAR T cells were apparent, and no toxicological changes of significance were seen. Serum biochemical parameters were analysed on days 28 and 56 after administration (**Figure 3**). On both days 28 and 56, the liver function indices, ALT and AST, were higher in the model control group and the normal T-cell group than in the negative control group, indicating hepatotoxicity caused by the cancer cells. On the other hand, the ALT and AST levels in the BCMA CAR-T treatment groups were significantly lower than in the model control group and normal T-cell group on day 28 (*P* < 0.01), and the ALT and AST levels in the BCMA CAR-thigh-dose group were similar to the negative control group and significantly lower than in the normal T-cell group (the model control animals died so that no comparison could be made) on day 56 (*P* < 0.01), suggesting that the BCMA CAR-T treatment can reduce hepatotoxicity in the MM-bearing mice.

**Figure 5:**
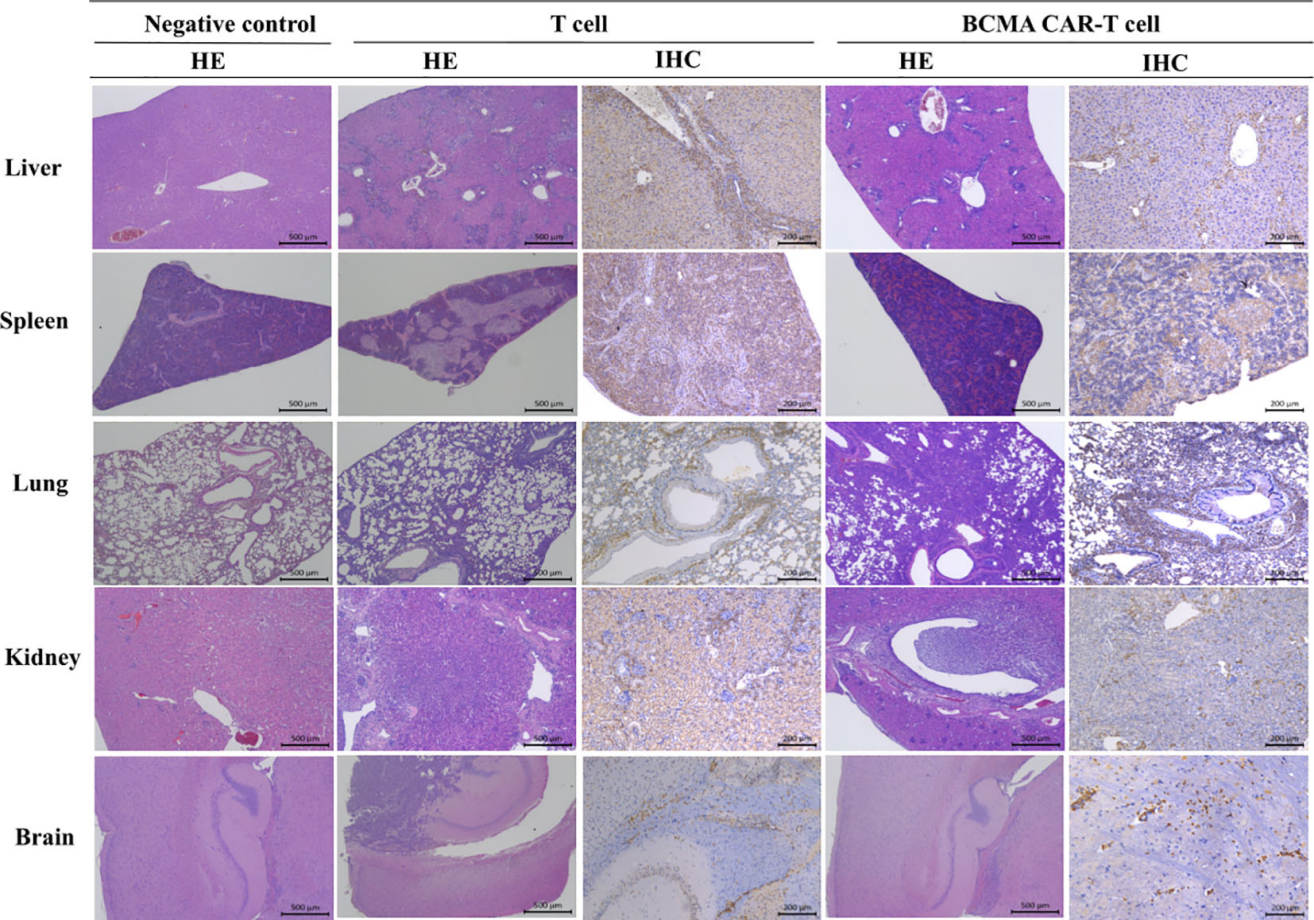
Histopathological changes in B-NDG mice after injection of T cells and BCMA CAR T cells (HE staining results and immunohistochemistry results. In this figure, mixed cell aggregates were observed in several tissues and organs of B-NDG mice, and the HE results showed the presence of T cells or CAR T cells around individual organs such as liver (500×), spleen (500×), kidney (500×), lung (500×), and brain (500×) in both T-cell control and BCMA CAR-T–treated groups. Infiltrative damage of inflammatory cells. In the immunohistochemical results, these inflammatory cells could be found to be mainly monocytes. At the end of the treatment period, the BCMA CAR T-cell treatment group showed less damage to tissues and organs than the T-cell control group, and no significant abnormal changes were observed in the rest of the organs and tissues.

On day 56, all cancer-bearing animals had died, and autopsy of these animals revealed varying degrees of cancer cell infiltration in several organs (sternum, femoral bone marrow, kidney, etc.) and cancer cell aggregation around the tissues (**Figure 5**). The fluctuations in ALT and AST levels could be attributed to the formation of MM in the liver, the aggregation of mixed cells, or the pharmacological effects of BCMA CAR-T *in vivo*. In the model control group, elevated ALT and AST levels on day 28 were associated with cancer cell load in the liver. However, ALT and AST were reduced in animals in the BCMA CAR-T treatment groups (**Figure 3**), suggesting an inhibitory effect on MM cells and thus reduced hepatic injury. These data suggest that cancer relapse occurred in the later stages even with the BCMA CAR T-cell treatment, while BCMA CAR T cells in the high dosage group were more durable resulting in delayed cancer recurrence.

In addition, on day 28, CK, Cl^+^ and GLDH levels were significantly lower in male animals (*P* < 0.05 or 0.01) and BUN, CK, K+, Cl+, and GLDH levels were significantly lower in female animals (*P* < 0.05 or 0.01) in the BCMA CAR-T–treated groups than the model control group. However, these parameters are in the normal background range, suggesting that restorative changes occurred in the hematological and biochemical parameters in MM-bearing mice treated with in the BCMA CAR T-cells, consistent with therapeutic effects. Based on these results, we conclude that none of the above haematological and serum biochemical parameters showed toxicologically significant changes after dosing.

Blood leukocytes and bone marrow cell-related indexes were analyzed in the mice on day 28 and day 56 after administration (**Tables 1**–**4**). Most of the leukocyte lineages (e.g., WBC, NEUT#, LYMPH#, MONO#, BASO#, MONO%, LYMPH%, and BASO%, in males and/or females) were higher in the model group mice than in the negative control group (*P* < 0.05, *P* < 0.01) (**Tables 1** and **2**). This hints at the occurrence of MM. On day 28, the BCMA CAR T-cell-treated groups and the T-cell group had reduced WBC levels compared to the model group, although the reduction was slight and the levels were still higher than the negative control group (*P* < 0.05, *P* < 0.01). However, the elevated neutrophils (NEUT#, NEUT%) in the BCMA CAR T-cell-treated groups were more similar to those in the negative control mice than the T-cell control group, possibly because the BCMA CAR T cells exerted a better therapeutic effect than normal T cells. On day 56, the WBC levels and the percentages of leukocytes (NEUT#, LYMPH#, LYMPH%) in the blood of the mice in the T-cell control and BCMA CAR T-cell-treated groups were elevated, and the NEUT% was decreased compared to the negative control group; however, the changes of these indicators in the BCMA CAR-T high group were less than those in the BCMA CAR-T low-dose group and T-cell control group. Most of the other leukocyte indicators in the blood of the mice in the BCMA CAR T-cell and normal T-cell–treated groups were not significantly different from those of the negative control group, and it could be seen that the number of leukocytes in the mice in the BCMA CAR-T high-dose group was more like that of the negative control group. Therefore, we believe that the observed phenomena reflect a therapeutic effect of BCMA CAR T-cells and not a toxicological change.

**Table 1:** Summary of results of BCMA CAR T cells on leukocyte sorting count indexes in the blood of multiple myeloma xenograft mouse model (male, days 28 and 56 days).

Time point	Group	*n*	WBC (10^9^/L)	NRBC#	NEUT# (10^9^/L)	LYMPH# (10^9^/L)	MONO# (10^9^/L)	EO# (10^9^/L)	BASO# (10^9^/L)	NRBC %	NEUT (%)	LYMPH %	MONO %	EO %	BASO %
4 weeks	Control	5	0.52 ± 0.21	0.00 ± 0.01	0.43 ± 0.19	0.05 ± 0.03	0.04 ± 0.02	0.00 ± 0.00	0.00 ± 0.00	0.8 ± 1.2	80.8 ± 7.6	10.7 ± 7.4	8.3 ± 2.6	0. 3 ± 0.6	0.0 ± 0.0
	Model	5	2.47 ± 1.42*	0.01 ± 0.01	1.37 ± 0.69*	0.69 ± 0.56	0.40 ± 0.36	0.00 ± 0.00	0.01 ± 0.01*	0.5 ± 0.5	62.6 ± 21.4	23.2 ± 15.1	13.7 ± 7.5	0.1 ± 0.2	0.5 ± 0.4*
	T cells	5	1.52 ± 0.45**	0.01 ± 0.01	1.23 ± 0.36**	0.17 ± 0.07**	0.11 ± 0.03**	0.01 ± 0.01	0.00 ± 0.00#	0.7 ± 0.4	80.9 ± 1.5	11.0 ± 2.0	7.5 ± 2.3	0.6 ± 0.4#	0.0 ± 0.0#
	BCMA CAR-T (5 × 10^7^)	5	1.45 ± 0.44**	0.01 ± 0.01	1.15 ± 0.23**	0.08 ± 0.05	0.21 ± 0.22	0.01 ± 0.00	0.00 ± 0.00#	0.4 ± 0.4	81.2 ± 9.0	5.7 ± 3.7	12.5 ± 8.3	0.6 ± 0.4#	0.0 ± 0.0#
	BCMA CAR-T (5 × 10^8^)	5	1.51 ± 0.39**	0.01 ± 0.00*	1.16 ± 0.35**	0.15 ± 0.07*	0.19 ± 0.05**△	0.01 ± 0.01*#	0.00 ± 0.00#	0.7 ± 0.2	76.1 ± 6.9	10.3 ± 5.4	12.9 ± 3.0*△	0.8 ± 0.5#	0.0 ± 0.0#
8 weeks	Control	5	2.97 ± 2.98	0.03 ± 0.04	1.59 ± 0.97	0.05 ± 0.05	0.20 ± 0.26	0.01 ± 0.01	0.00 ± 0.00	0.9 ± 0.7	92.7 ± 3.6	1.5 ± 0.6	5.5 ± 3.7	0.3 ± 0.3	0.0 ± 0.0
	T cells	2	2.22 ± 0.10	0.02 ± 0.01	1.61 ± 0.40	0.40 ± 0.36	0.21 ± 0.13	0.01 ± 0.01	0.01 ± 0.01	0.7 ± 0.4	72.8 ± 21.4	17.5 ± 15.5	9.4 ± 5.3	0.2 ± 0.3	0.2 ± 0.3
	BCMA CAR-T (5 × 10^7^)	3	4.05 ± 1.92	0.01 ± 0.01	3.45 ± 1.62	0.20 ± 0.19	0.39 ± 0.16	0.01 ± 0.01	0.00 ± 0.00	0.2 ± 0.3	85.3 ± 0.7*	4.5 ± 2.3	10.0 ± 2.3	0.2 ± 0.3	0.0 ± 0.0
	BCMA CAR-T (5×10^8^)	4	3.12 ± 2.75	0.03 ± 0.02	1.06 ± 0.86	0.57 ± 0.76	0.59 ± 0.53	0.01 ± 0.01	0.01 ± 0.01	1.3 ± 1.5	65.0 ± 15.3*	16.6 ± 13.1	17.9 ± 3.5**	0.4 ± 0.7	0.1 ± 0.2

Compared with control group, ^*^ *P* < 0.05, ^**^ *P* < 0.01; compared with model group, ^#^ *P* < 0.05, ^##^ *P* < 0.01; compared with T-cell group, ^△^ *P* < 0.05, ^△△^ *P* < 0.01. WBC, white blood cell count; NEUT#, absolute neutrophil value; BASO#, absolute basophil value; BASO%, basophil percentage; LYMPH#, absolute lymphocyte value; LYMPH%, lymphocyte percentage; NEUT%, neutrophil percentage. WBC, white blood cell count; NEUT#, absolute value of neutrophils; BASO#, absolute value of basophils; BASO%, percentage of basophils; LYMPH#, absolute value of lymphocytes; LYMPH%, percentage of lymphocytes; NEUT%, percentage of neutrophils; MONO#, absolute value of monocytes; NRBC#, absolute value of nucleated cells; EO%, percentage of eosinophils.

**Table 2:** Summary of results of BCMA CAR T cells on leukocyte sorting count indexes in the blood of multiple myeloma xenograft mouse model (female, days 28 and 56 days).

Time point	Group	*n*	WBC (10^9^/L)	NRBC#	NEUT# (10^9^/L)	LYMPH# (10^9^/L)	MONO# (10^9^/L)	EO# (10^9^/L)	BASO# (10^9^/L)	NRBC %	NEUT (%)	LYMPH %	MONO %	EO %	BASO %
4 weeks	control	5	0.39 ± 0.18	0.00 ± 0.01	0.38 ± 0.17	0.00 ± 0.00	0.01 ± 0.01	0.00 ± 0.00	0.00 ± 0.00	0.7 ± 1.0	97.3 ± 2.5	0.3 ± 0.8	1.4 ± 1.9	1.0 ± 2.2	0.0 ± 0.0
	model	5	1.38 ± 0.85	0.01 ± 0.01	0.63 ± 0.44	0.47 ± 0.29*	0.27 ± 0.25	0.00 ± 0.01	0.01 ± 0.01	0.7 ± 0.9	47.9 ± 14.3**	33.7 ± 4.5**	18.1 ± 12.1	0.1 ± 0.2	0.3 ± 0.3
	T cells	5	1.34 ± 0.78	0.02 ± 0.02	0.92 ± 0.49*	0.21 ± 0.16*	0.22 ± 0.15*	0.00 ± 0.00	0.00 ± 0.01	1.8 ± 2.0	69.2 ± 9.5**#	14.8 ± 6.8**##	15.7 ± 2.6**	0.0 ± 0.0	0.3 ± 0.4
	BCMA CAR-T (5 × 10^7^)	5	1.17 ± 0.55*	0.02 ± 0.01*#	1.13 ± 0.52*	0.00 ± 0.01#	0.03 ± 0.05	0.00 ± 0.00	0.00 ± 0.00	1.7 ± 0.7	96.9 ± 3.1##	0.3 ± 0.5##	2.6 ± 2.6	0.1 ± 0.3	0 ± 0.0
	BCMA CAR-T (5 × 10^8^)	5	1.37 ± 0.48**	0.00 ± 0.01	1.07 ± 0.42**	0.13 ± 0.06**	0.16 ± 0.08**	0.01 ± 0.01△	0.00 ± 0.00	0.3 ± 0.4	77.6 ± 7.0**##	10.1 ± 4.8*##	11.5 ± 3.5**	0.7 ± 0.5△	0.0 ± 0.0
8 weeks	control	5	0.51 ± 0.18	0.01 ± 0.00	0.47 ± 0.19	0.02 ± 0.02	0.02 ± 0.01	0.00 ± 0.01	0.00 ± 0.00	2.1 ± 0.7	90.1 ± 6.3	0.9 ± 0.7	4.3 ± 2.4	1.0 ± 1.4	0.0 ± 0.0
	T cells	5	2.14 ± 2.40	0.05 ± 0.08	1.16 ± 1.02	0.64 ± 0.96	0.33 ± 0.43	0.00 ± 0.00	0.01 ± 0.01	1.7 ± 1.6	61.4 ± 12.5**	24.4 ± 10.2**	14.0 ± 2.5**	0.0 ± 0.1	0.2 ± 0.2
	BCMA CAR-T (5 × 10^7^)	5	3.65 ± 2.99	0.01 ± 0.01	3.19 ± 2.72	0.19 ± 0.12*	0.26 ± 0.22	0.02 ± 0.04	0.00 ± 0.00	0.6 ± 0.5**	82.9 ± 16.8	9.2 ± 13.1	7.4 ± 3.9	0.4 ± 0.6	0.0 ± 0.0
	BCMA CAR-T (5 × 10^8^)	5	1.13 ± 0.94	0.03 ± 0.04	0.94 ± 0.79	0.11 ± 0.21	0.07 ± 0.10	0.00 ± 0.00	0.00 ± 0.00	3.5 ± 3.3	89.0 ± 16.1△	6.4 ± 11.3△	4.5 ± 4.9△△	0.1 ± 0.2	0.0 ± 0.0

Compared with control group, ^*^ *P* < 0.05, ^**^ *P* < 0.01; compared with model control, ^#^ *P* < 0.05, ^##^ *P* < 0.01; compared with T-cells group, ^△^ *P* < 0.05, ^△△^ *P* < 0.01. WBC, white blood cell count; NEUT#, absolute neutrophil value; BASO#, basophil absolute value; BASO%, basophil percentage; LYMPH#, absolute value of lymphocytes; LYMPH%, lymphocyte percentage; NEUT%, neutrophil percentage. MONO#, absolute monocyte value; NRBC#, absolute nucleated cell value; EO%, eosinophil percentage.

**Table 3:** Summary of the results of BCMA CAR T-cell injection on the classification index of bone marrow cells in the blood of multiple myeloma xenograft mice model (male, days 28 and 56 days).

Time point	Group	*N*	WBC (10^9^/L)	RBC (10^12^/L)	Acaryote (%)	NEUT (%)	LYMPH (%)	MONO (%)	EO (%)	BASO (%)
4 weeks	Control	10	20.34 ± 2.48	0.02 ± 0.00	46.42 ± 7.36	44.2 ± 6.7	27.6 ± 5.8	23.6 ± 2.0	1.0 ± 0.2	3.6 ± 0.8
	Model	12	20.42 ± 7.12	0.01 ± 0.00**	36.17 ± 10.50*	82.3 ± 8.4**	7.4 ± 10.0**	10.0 ± 4.6**	0.1 ± 0.0**	0.2 ± 0.3**
	T cells	10	24.91 ± 9.35	0.02 ± 0.01##	44.29 ± 13.82	45.6 ± 13.9##	27.6 ± 5.1##	22.4 ± 8.5##	0.9 ± 0.6##	3.5 ± 2.5##
	BCMA CAR-T(5 × 10^7^)	10	28.06 ± 5.45**#	0.02 ± 0.00##	43.07 ± 4.57	41.9 ± 5.7##	27.9 ± 4.1##	24.8 ± 3.1##	1.2 ± 0.5##	4.2 ± 1.1##
	BCMA CAR-T(5 × 10^8^)	10	24.07 ± 4.43*	0.02 ± 0.00##	45.83 ± 4.89#	42.0 ± 4.0##	28.7 ± 2.3##	24.1 ± 2.4##	1.2 ± 0.5##	4.1 ± 0.6##
8 weeks	control	9	33.13 ± 3.67	0.02 ± 0.00	38.91 ± 4.63	42.5 ± 5.6	28.1 ± 4.1	24.7 ± 1.8	0.8 ± 0.3	3.8 ± 0.9
	T cells	6	15.42 ± 10.01**	0.01 ± 0.01**	50.71 ± 18.87	50.9 ± 10.0	28.9 ± 11.0	18.0 ± 2.4**	0.7 ± 0.2	1.5 ± 0.5**
	BCMA CAR-T(5 × 10^7^)	6	12.65 ± 5.08**	0.02 ± 0.01	56.84 ± 8.08**	52.3 ± 17.8	29.0 ± 11.2	16.0 ± 7.7*	0.9 ± 0.4	1.8 ± 1.5**
	BCMA CAR-T(5 × 10^8^)	9	22.33 ± 6.66**	0.02 ± 0.00	44.73 ± 9.64	44.9 ± 8.1	29.4 ± 6.1	22.2 ± 4.8	0.9 ± 0.4	2.7 ± 1.2△

WBC, white blood cell count; RBC, red blood cell count; Acaryote, nucleated cells; NEUT%, neutrophil percentage; LYMPH%, lymphocyte percentage; MONO%, monocyte ratio; EO%, eosinophil ratio; BASO%, basophil ratio. Compared with negative control, ^*^ *P* < 0.05, ^**^ *P* < 0.01; compared with model group, ^#^ *P* < 0.05, ^##^ *P* < 0.01; compared with human T-cell group, ^△^ *P* < 0.05, ^△△^ *P* < 0.01. Fourth week and eighth week indicate that these data were obtained after days 28 and day 56.

**Table 4:** Summary of the results of BCMA CAR T-cell injection on the classification index of bone marrow cells in the blood of multiple myeloma xenograft mice model (female, days 28 and 56).

Time point	Group	*n*	WBC (10^9^/L)	RBC (10^12^/L)	Acaryote (%)	NEUT (%)	LYMPH (%)	MONO (%)	EO (%)	BASO (%)
4 weeks	Control	10	16.05 ± 4.01	0.02 ± 0.01	49.31 ± 5.89	39.2 ± 3.3	29.8 ± 2.0	25.8 ± 1.7	0.8 ± 0.2	4.5 ± 0.7
	Model	8	15.01 ± 4.14	0.01 ± 0.01	47.31 ± 15.00	87.8 ± 4.7**	8.6 ± 4.6**	3.4 ± 1.4**	0.1 ± 0.0**	0.2 ± 0.0**
	T cells	10	28.27 ± 6.65**##	0.02 ± 0.01	36.98 ± 9.43**	41.1 ± 6.0##	30.3 ± 5.1##	24.3 ± 4.5##	0.7 ± 0.3##	3.6 ± 1.3##
	BCMA CAR-T(5 × 10^7^)	10	23.37 ± 6.40##	0.02 ± 0.01#	45.84 ± 3.88	38.8 ± 2.1##	29.4 ± 1.2##	26.0 ± 1.0##	0.9 ± 0.2##	5.0 ± 0.5##
	BCMA CAR-T(5 × 10^8^)	10	20.74 ± 4.45#△△	0.02 ± 0.00##	50.66 ± 5.25△△	38.1 ± 6.2##	29.8 ± 4.7##	26.2 ± 3.1##	0.9 ± 0.3##	4.9 ± 0.9##△
8 weeks	Control	10	20.67 ± 4.41	0.02 ± 0.01	47.47 ± 6.74	32.9 ± 3.7	30.5 ± 2.6	29.1 ± 3.2	0.8 ± 0.2	6.6 ± 1.0
	T cells	9	14.73 ± 6.49*	0.01 ± 0.01*	49.03 ± 12.96	45.0 ± 8.0**	29.5 ± 3.1	22.5 ± 5.6**	0.4 ± 0.1**	2.6 ± 1.8**
	BCMA CAR-T(5 × 10^7^)	9	16.20 ± 5.35	0.01 ± 0.01	46.66 ± 12.36	41.7 ± 11.2	32.2 ± 8.9	22.1 ± 8.6	0.7 ± 0.3	3.3 ± 2.1
	BCMA CAR-T(5 × 10^8^)	10	18.23 ± 3.80	0.02 ± 0.01	44.42 ± 7.94	41.3 ± 9.6	27.8 ± 8.1	25.8 ± 3.6	0.8 ± 0.4△	4.4 ± 1.6△

WBC, white blood cell count; RBC, red blood cell count; Acaryote, nucleated cells; NEUT%, neutrophil percentage; LYMPH%, lymphocyte percentage; MONO%, monocyte ratio; EO%, eosinophil ratio; BASO%, basophil ratio. Compared with negative control, ^*^ *P* < 0.05, ^**^ *P* < 0.01; compared with model group, ^#^ *P* < 0.05, ^##^ *P* < 0.01; compared with human T-cell group, ^△^ *P* < 0.05, ^△△^ *P* < 0.01. Fourth week and eighth week indicate that these data were obtained on day 28 and day 56.

The results of bone marrow cell sorting in mice (**Tables 3** and **4**) showed that the leukocyte count (e.g., WBC) was higher in the BCMA CAR T-cell treatment groups and the T-cell group than in the negative control group on day 28, but we do not consider this to be a toxicological response. This is because the leukocyte classification percentages such as LYMPH%, MONO%, EO%, and BASO% were elevated in all three dosing groups compared with the model group, and the values were close to those of the negative control group. We speculate that the elevated leukocyte levels in mice reflected T cells, which maintained the balance of various types of leukocyte levels in the bone marrow of the mice, while the BCMA CAR T cells showed greater therapeutic effects. However, on day 56, the leukocytes such as WBC and leukocyte classification percentages MONO%, EO%, BASO% decreased and NEUT% increased in the T-cell control and BCMA CAR T-cell groups compared with the negative control group (*P* < 0.05, *P* < 0.01). We could see a spread of cancer cells in some mouse tissues based on the histopathological results on day 56. Therefore, we consider it likely that cancer recurrence in the tissues of the mice led to the disruption of the leukocyte levels *in vivo*; however, the changes in the leukocyte indexes were less in the BCMA CAR T-cell high-dose group compared to the T-cell group with the same number of T cells as the BCMA CAR T-cell high-dose groups, reflecting the better inhibition of cancer recurrence in the BCMA CAR T-cell high-dose group. Together, these data suggest that the changes in the bone marrow indicators were not due to BCMA CAR T-cell toxicity.

### No significant cytokine storm in the BCMA CAR T-cell-treated B-NDG mice

**Figure 6:**
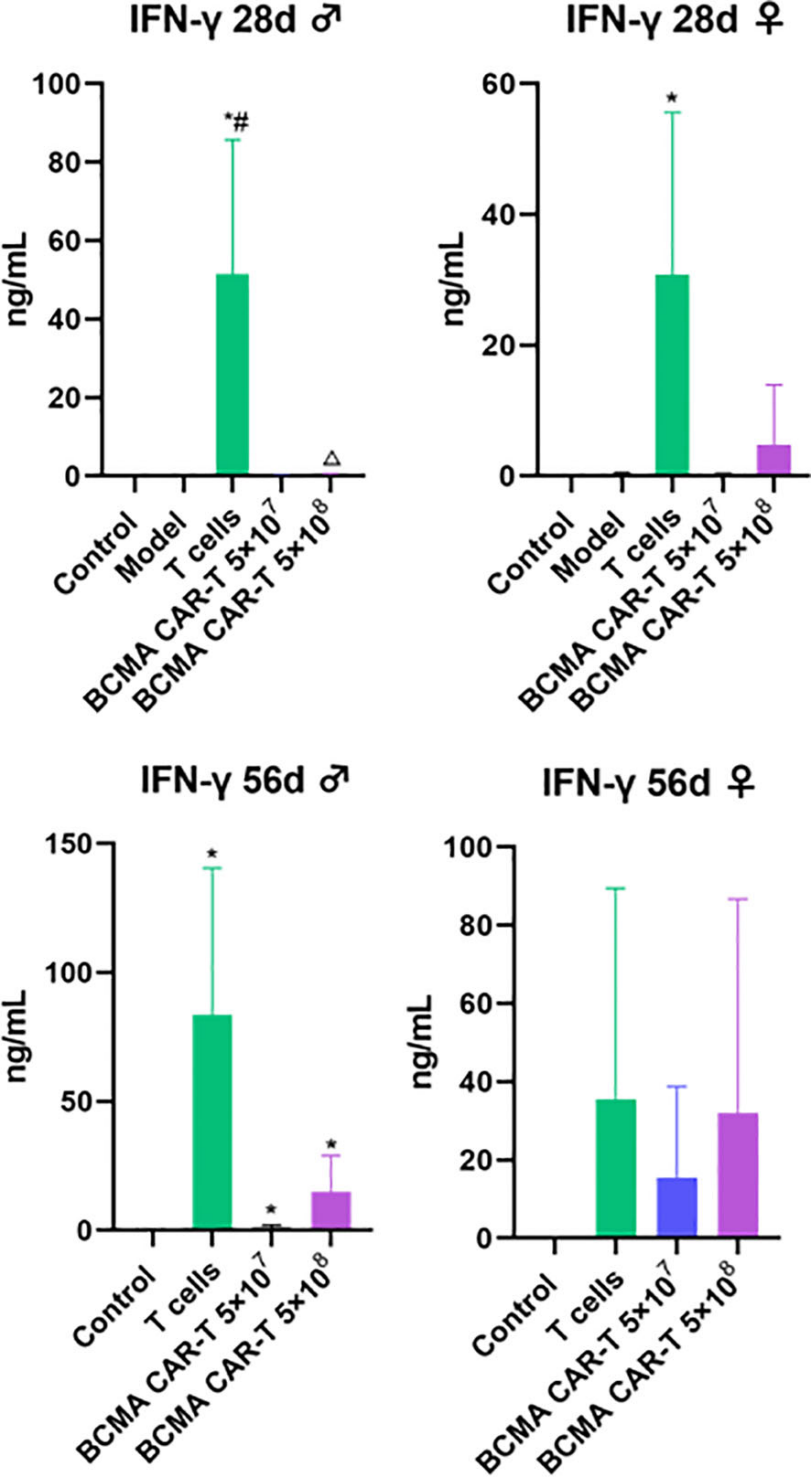
Human cytokine levels in BCMA CAR T-cell-injected xenograft model mice (control *n* = 5, model *n* = 5, T-cells *n* = 5, rest of animals *n* = 10). Exponentially elevated IFN-γ, and the rest of cytokines <1 ng, data not shown. IFN-γ, interferon gamma; TNF, tumor necrosis factor; IL, interleukin. Compared with control group, *P < 0.05; compared with model group, ^#^ *P* < 0.05; compared with T cell group, ^△^ *P* < 0.05.

We measured human-derived cytokines and T-lymphocyte subsets on days 28 and 56 after the BCMA CAR T-cell injection to assess immuno-toxicity in the mice. Human-derived cytokines IFN-γ, IL-2, IL-4, IL-6, IL-12p70, IL-10, and TNF-α were all measured (**Figure 6**). On day 28, the T-cell control group showed a geometric increase in IFN-γ levels, suggesting that the mice were producing an intense cytokine storm, possibly related to the GVHD response. The remaining cytokine levels did not show significant fluctuations (< 1 ng/mL), suggesting that the administration of the BCMA CAR-T or normal T cells did not cause toxicologically significant changes in these indicators, and therefore the data of the remaining cytokines are not presented. Although the IFN-γ levels in the BCMA CAR-T–treated groups had a certain degree of elevation and showed the same cancer cell-killing effects, the factor levels did not show a geometric increase and all other indicators in the animals were normal (**Figure 6**). The elevated cytokines were judged to result from cancer cell killing by the BCMA CAR T cells, and the data suggest that the probability of a dramatic clinical cytokine storm is extremely low.

**Figure 7:**
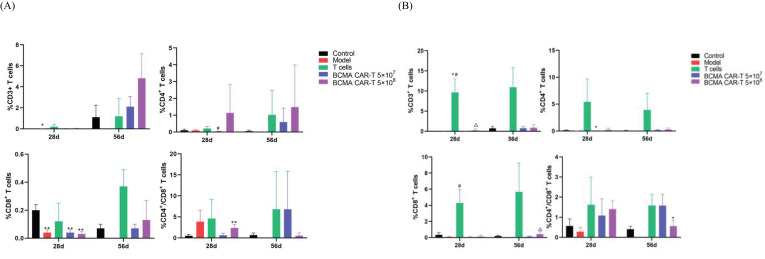
T-lymph subpopulation levels of BCMA CAR T-cell injection in xenograft model mice (n=10 for control group, n=9 for model group n=10 for the T-cell group, n=20 for the rest animals). **(A)** indicates cytokines in male animals and **(B)** indicates cytokines in female animals. 28d and 56d refer to days 28 and 56 after drug administration, respectively. Compared with control group, * *P*<0.05, ** *P*<0.01; compared with model group, ^#^ *P*<0.05; compared with T cells group, ^△^ *P*<0.05. **(A)** CD4^+^ and CD8^+^ were higher than control group (*P<0.05, **P<0.01); **(B)** CD4^+^ was higher than control group (*P<0.05, **P<0.01); CD3^+^ and CD8^+^ were lower than Tcell group (*P<0.05,**P<0.01); CD4^+^/CD8^+^ was higher than control group (*P<0.05).

We also measured human-derived T-lymphocyte subpopulations on days 28 and 56 (**Figure 7**). The results showed that *h*CD3^+^, *h*CD4^+^, and *h*CD8^+^T lymphocytes were rarely detectable in the negative control and model control groups, reflecting the assay’s specificity. We could detect low levels of *h*CD3^+^, *h*CD4^+^, and *h*CD8^+^ T lymphocytes in peripheral blood mononuclear cells (PBMC) isolated from the mice whole blood in the T-cell control group and BCMA CAR T-cell treatment groups. However, the percentages of *h*CD3^+^, *h*CD4^+^, and *h*CD8^+^ T lymphocytes were higher in the T-cell control group than in the BCMA CAR T-cell treatment groups (*P* < 0.05), showing that the majority of human-derived T cells in the T-cell control group were distributed in the peripheral blood circulation, whereas the BCMA CAR T-cell groups only had a higher percentage of *h*CD3^+^whilelower percentages of *h*CD4^+^ and *h*CD8^+^ were found in the peripheral blood. Combined with the histopathological findings, the T-cell control group showed inflammatory infiltration of T cells around the peripheral blood vessels of the tissues and organs, suggesting that human-derived T cells are more likely to be distributed in non-cancer–targeted tissues and organs as well as in the peripheral blood, and therefore the T-cell control group had a more intense GvHD response than the BCMA CAR T-cell treatment groups, ultimately resulting insignificant infiltration of T cells and inflammatory cells in various tissues and organs in addition to cancer-targeted tissue (**Figure 5**). The high percentage of *h*CD3^+^ and low percentages of *h*CD4^+^ and *h*CD8^+^ in the peripheral blood in the BCMA CAR T-cell treatment groups, combined with the cancer killing effects of *h*CD4^+^ and *h*CD8^+^, suggest that BCMA CAR T cells have a better effect and cancer antigen recognition ability compared to normal T cells. These results suggest that BCMA CAR T cells can better target the cancer cells and reduce the probability of cytokine outbreaks, and that the alterations in the indices did not indicate toxicological significance.

### Mild histopathological alterations in MM mice and the biodistribution of CAR genes consistent with cancer cell metastasis pathways after the CAR T-cell treatment

Histopathology showed varying degrees of cancer cell infiltration or accumulation of cancer cells around the tissues of both the dead and surviving animals, such as the bone marrow of the sternum and femur, kidneys, lungs, spleen, liver, brain, and so forth (**Figure 5**). Cancer cell infiltration in these areas suggested that they were focal metastatic sites. In the BCMA CAR T-cell treatment groups and the T-cell control group, it could be observed that a mixture of human-derived T cells, cancer cells and inflammatory cells infiltrated into several organs and tissues (including liver, spleen, lung, and kidney) in some animals in both the T-cell control group and the BCMA CAR T-cell high-dose group, and the infiltration became more significant as time increased.

The tissue distribution results showed that the males in the BCMA CAR-T low-dose group had the highest levels in the brain, followed by the kidney, lung, bone marrow, pancreas, spleen and epididymis (**Figure 5**). Many BCMA CAR Tcells were distributed in the brain, but no organ damage was seen on pathological examination. In the females of the BCMA CAR-T low-dose group, the level of the BCMA CAR T cells in the kidney was the highest, followed by bone marrow, small intestine, uterus, lung, stomach, spleen and brain. In the males of the high BCMA CAR-T high-dose group, the levels were highest in the bone marrow, followed by the brain, spleen, lungs, epididymis and kidneys. In the females of the high BCMA CAR-T dose group, the levels were highest in the uterus, followed by the lungs, spleen, bone marrow, kidneys, liver, and spinal cord (**Figure 5**).

**Figure 8:**
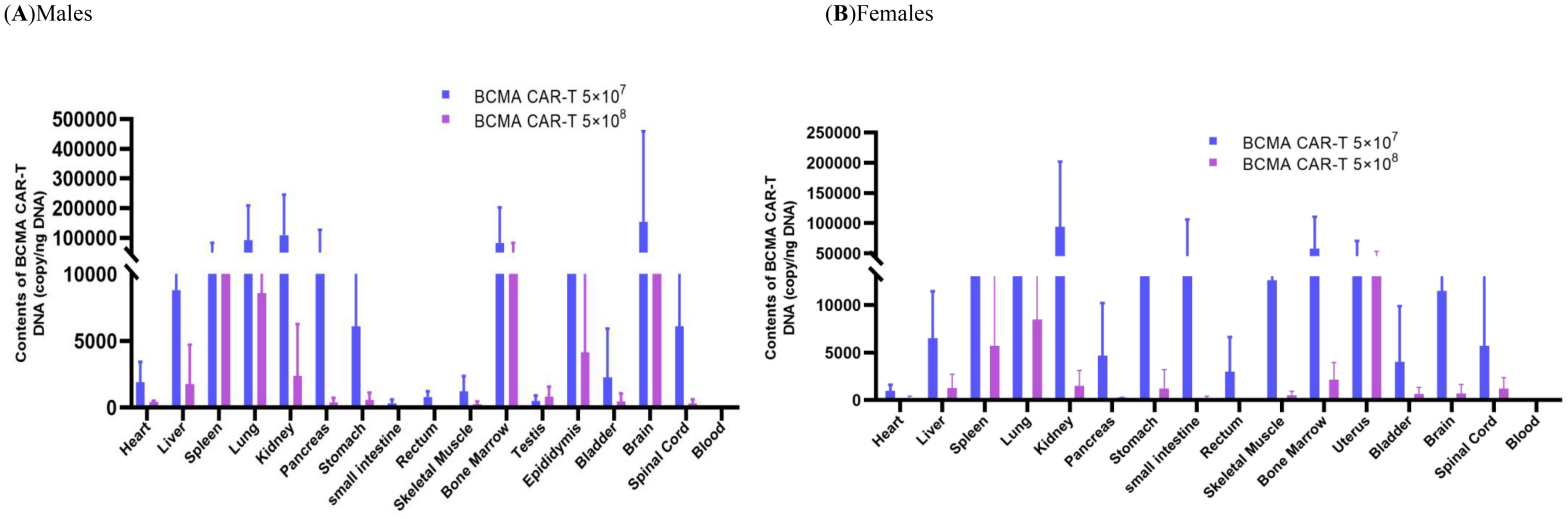
Biodistribution in the xenograftmouse model administered with BCMA CAR T-cell injection. Biodistribution of B-NDG mice. **(A)** males and **(B)** females administered with BCMA CAR T-cell injection in various tissues after day 56 (*n* = 5, per group). The DNA of BCMA CAR-T was widely distributed in B-NDG mice after a single administration. **(A)** Peak BCMA CAR-T DNA content in the brain of male animals on day 56 after administration, followed by kidney, lung, bone marrow, pancreas, and spleen; **(B)** Peak BCMA CAR-T DNA content in the kidney of female animals after day of administration, followed by bone marrow, small intestine, uterus and lung. The lower levels of BCMA CAR-T DNA obtained from the peripheral blood of **(A)** and **(B)** animals indicate that the drug underwent targeted tissue transfer.

The histopathological and tissue distribution results suggest that normal T cells and BCMA CAR T cells are distributed along with the blood to all major organs, mainly in the kidney, lung, bone marrow, and immune organs; however, the distribution of BCMA CAR T cells is highly consistent with the metastatic pathway of MM cancer cells and does not cause excessive immune responses (**Figure 8**). We therefore consider it likely that the BCMA CAR-T cells have a therapeutic effect on the cancer metastasis targeting tissues while avoiding side effects from off-targeting. Combined with the data from the immunogenicity studies, we could not find any evidence of excessive immune responses or cytokine storm associated with the administration of BCMA CAR T cells, which are modified using the patient’s own cells. Therefore, we believe that the potential for immuno-toxicity of the BCMA CAR T cells in the future clinical treatment is low.

## Discussion

Today, the incidence of MM remains a concern, and although significant advances in the treatment of MM have been made over the past decade or so, with significant increases in survival rates, ultimately most patients cannot escape drug resistance and relapse (29, 30). CAR T cells are the most promising drug for curing patients with relapsed/refractory MM due to their powerful specificity. There have also been many surprising results from studies of CAR-T in haematological malignancies, for example, CAR T cells have shown great success in other haematological malignancies such as acute lymphoid leukaemia (31, 32). CAR T-cell products have therefore started to become the focus of research in the past few years, with many CAR T-cell products entering preclinical and clinical trials, and a large number of preclinical studies have demonstrated the powerful anti-cancer activities of CAR T cells. However, in the clinical trial phase, patients who received CAR T-cell infusions often experienced toxic side effects (mainly CRS, NXT) resulting in death in the vast majority of cases, so severe toxicity remains a major limiting factor for CAR T-cell therapy to cure patients at present.

There have been few reports on the clinical application of BCMA CAR-T so far, and the clinical cell dosage is 2 × 10^6^–10 × 10^6^ cells/kg. To investigate the potential toxicity of different doses of BCMA CAR-T in preclinical settings, we selected B-NDG severe immune deficient mice, and based on the available clinical data, we determined to use the BCMA CAR T cells in animals at doses of 5 × 10^7^ and 5 × 10^8^ cells/kg, approximately five and 50 times the clinical dosage, respectively. Because CAR T cells have the ability to proliferate *in vivo*, the number of cells produced by proliferation *in vivo* appears to be more important than the number of cells initially infused into the patient. Our data suggest that a better efficacy may often be obtained with higher dosages, which is consistent with the findings of previous studies (33–35). However, obtaining good efficacy must be accompanied by playing attention to safety issues to avoid serious adverse effects from the associated toxic reactions that could compromise efficacy. Our results show that in the BCMA CAR-T high-dose group, the tissue damage and other toxicities caused by the mixed cell infiltration in the organs were less than those in the T-cell group.

In toxicity studies, changes in body weight, food intake, haematology, and serum biochemistry are generally the chemical indicators that should be monitored as a priority after drug administration (36–38). CAR T-cell therapy is immunotherapy, immuno-toxicity derived from an overloading immune response is another important concern. In this delayed toxicity study, only a few animals died in the treatment groups, whereas all mice died in the model control group. The number of mice died in the high- and low-dose groups of BCMA CAR T cells was 1 and 2, respectively; however, the T-cell control group had five cases. The five death cases and there duction of the body weight in the remaining animals in the T-cell group were possibly due to the spread of cancer cells in the B-NGD mice. In the BCMA CAR-T treatment group, most animals showed the cancer targeting nature of the BCMA CAR T cells, as a high number of BCMA CAR T cells have in filtrated the focal cancer cell targeted organs with little off-targeting in tissue distribution studies and histopathological studies. The body weight and food intake of B-NDG mice fluctuated up and down to a certain extent (especially in females) caused by the cancer cells before administration, and after administration, both body weight and food intake increased and even returned to a normal diet level in the BCMA CAR-T high dose group animals.

Haematological indicators are essential for determining the toxicity of drugs. In the BCMA CAR T-cell treatment groups, the erythrocyte lineage, leukocyte lineage, platelets, and coagulation function showed an abnormal state before the treatment, but the relevant indicators improved to different degrees after the administration of the BCMA CAR T cells, and the clinical symptoms were relatively mild after the administration. On day 56 after administration, there was almost no significant difference in most of the indicators compared to the negative control group, which indicates that the abnormal haematological indicators normalized after a period of treatment, and the other indicators did not change to a significant extent and therefore were not considered toxicologically significant. Similarly, we examined the liver function, kidney function and electrolytes, and found that there were almost no significant differences in AST, ALT, Ca^2+^, and K^+^ between the BCMA CAR T-cell groups or T-cell group compared to the negative control group, while these indicators were abnormal in the model mice. These results also indicate that the BCMA CAR T cells had certain therapeutic effects on the cancer animal model. Moreover, while various indicators of the nutritional status showed abnormality in the cancer-wasting mice, an improvement of nutritional status in the BCMA CAR T-cell high-dose group was more obvious than that in the low-dose group and T-cell control group.

In haematological malignancies, excessive activation and expansion of CAR T cells can directly lead to high levels of cytokine/chemokine secretion, and CRS is one of the serious and most potentially life-threatening toxicities of CAR T-cell therapy. Among these, the levels of IL-6 and IFN-γ are important safety indicators (39–41). Our data indicate that on day 28, only the T-cell group showed geometrically elevated levels of IFN-γ, suggesting that a cytokine storm due to GVHD may have been generated, while the BCMA CAR T-cell groups showed a controlled elevated IFN-γ, with no significant cytokine storm, and the remaining cytokines (including IL-6) were at a low level below 1 ng/ml. Therefore, we conclude that BCMA CAR T cells did not result in CRS during the treatment. Our data suggest that the risk of severe cytokine storm in the future clinical application of BCMA CAR T cells is extremely low, as BCMA CAR T cells from patients’ autologous cells will be used for transfusion, although close monitoring of cytokine level changes during clinical administration will still be necessary.

Tissue distribution and histopathological studies are integral parts of the efficacy and safety evaluation of BCMA CAR T cells, with the aims to identify the organs or tissues where CAR genes may reach and to evaluate the safety of CAR T cells in terms of tissue organ morphology (42). In the tissue distribution study, our data suggest that 56 days after administration, the distribution of BCMA CAR T cells is highly consistent with the pathway of MM cancer development and migration. In addition, we found that the level of BCMA CAR T cells in the whole blood decreased over time and the number of CAR copies in the high-dose group was lower than that in the low-dose group. This might have resulted from cancer metastatic recurrence leading to the proliferation of CAR T cells to exert anti-cancer effects.

The histopathological results showed that the target tissues of the cancer-bearing B-NDG mice in the T-cell control and BCMA CAR T-cell treatment groups had a mixed cellular infiltration of the human-derived T cells, cancer cells, and inflammatory cells, with the degree of change increased over time. It is clear that the BCMA CAR T-cell treatment group showed better therapeutic effects, and the mixed immune cell aggregation phenomena indirectly reflected the pharmacological effects of BCMA CAR T cells. This is consistent with the results of the tissue distribution study, but the tissue damages were more severe in human-derived T-cell group than in the high BCMA CAR T-cell dose group, suggesting that human-derived T cell cells may appear to have a non-targeted killing effect. All of the results illustrate that the CAR T cells reach the cancer site as cancer metastasizes, demonstrating strong targeting effects.

As an innovative immunotherapy, CAR-T therapy has shown remarkable results in the field of cancer treatment. However, with the emergence of its efficacy, the related adverse reactions should not be ignored. These adverse reactions can be divided into acute toxicity and long-term adverse reactions in terms of time limit. We summarize the adverse effects of several BCMA CAR-T products that are currently marketed or in the clinical trials. The long-term adverse reactions of BCMA CAR-T therapy are mainly B-cell depletion, hypogammaglobulinosis, infection, and cytopenia (**Table 5**). For example, in the phase 3 clinical trials of the marketed Idecabtagene Vicleucel, more than 80% of patients will develop CRS, most of which are grades 1–2, but some patients will experience more severe grade 3 or above reactions. In addition, about 15%–20% of patients will develop immune effector cell-associated neurotoxic syndrome (ICANS), which mainly presents mild symptoms. Cytopenia occurs in 85% of patients, and the risk of infection is high, especially early in treatment (43–45). Another marketed product, Ciltacabtagene Autoleucel, also has high adverse reactions. The incidence of CRS is close to 80%, of which 5%–10% of patients may develop severe CRS of grades 3–4. The incidence of ICANS is about 20%. Although most symptoms are mild and reversible, some patients may still experience cognitive decline and other problems. In addition, the risk of cytopenias and infection is equally significant with this product (46, 47). Adverse reactions such as CRS, ICANS, cytopenias, and infection are also common for products currently in phase I or II. From the current I/II data of P-BCMA-101, the incidence of CRS and ICANS of P-BCMA-101. It is relatively low and the symptoms are mild and mild in some patients (48). Although the CRS incidence rate of Zevorcabtagene Autoleucel is higher than 50%, most of them are grades 1–2 reactions, severe CRS is rare, and the incidence rate of ICANS is low (49). Although the adverse reactions of BCMA’s CAR T-cell therapy are more obvious, under standard treatment and monitoring, most reactions are controllable and can bring important clinical benefits to patients with MM. These reactions may be related to the sustained killing of normal B cells by CAR T cells, resulting in a weakened immune system, susceptibility to infection, and possible symptoms such as cytopenias. The most obvious acute adverse reactions in CAR-T therapy include CRS and immune effector cell-associated neurotoxicity syndrome. The occurrence of CRS is mainly due to the production of a large number of cytokines such as IL-6, IL-10, TNF-α, GM-CSF, and IFN-γ after CAR T-cell activation. The rapid release of these cytokines leads to a systemic inflammatory response, in which IL-6 plays a key role in inducing a strong immune response and the development of CRS. IFN-γ can induce the activation of immune cells, especially macrophages, further exacerbating the degree of CRS and neurotoxicity. On the one hand, cytokines such as TNF-α and IFN-γ in the blood can promote the development of neurotoxicity. On the other hand, CAR T cells themselves are also able to enter the cerebrospinal fluid and cause direct damage to the central nervous system.

**Table 5:** Products targeting BCMA CAR-T for the treatment of multiple myeloma and major clinical adverse reactions.

Name	Investigators	R&D progress	Pivotal clinical studies	Major adverse reactions and incidence	References
Ide-cel	Celgene Corp,	Launched	NCT02658929 (Phase I)	CRS (76%) Neurologic toxic effect (42%) Neutropenia (85%) Anemia (58%) Leukopenia (61%) Thrombocytopenia (58%) Lymphopenia (18%)	(43)
			NCT03361748 (Phase II)	CRS (84%) Neurologic toxic effect (18%) Neutropenia (91%) Anemia (70%) Leukopenia (42%) Thrombocytopenia (63%) Lymphopenia (27%)	(44)
			NCT03651128 (Phase III)	CRS (88%) Neurologic toxic effect (15%) Neutropenia (78%) Anemia (66%) Leukopenia (29%) Thrombocytopenia (54%) Lymphopenia (29%)	(45)
Cilta-cel	Janssen Research & Development and Legend Biotech	Launched	NCT03548207 (Phase Ib/II)	CRS (95%) Neurologic toxic effect (21%) Neutropenia (95%) Anemia (68%) Leukopenia (61%) Thrombocytopenia (60%) Lymphopenia (50%)	(46)
			NCT04181827 (Phase III)	CRS (76.1%) Neurologic toxic effect (20.5%) Neutropenia (89.9%) Anemia (54.3%) Thrombocytopenia (54.3%) Lymphopenia (22.1%) hypogammaglobulinemia (42.3%)	(47)
Equecabtagene Autoleucel	IASO Biotechnology and Innovent Biologics	Phase III	ChiCTR1800018137 (Phase I)	CRS (94.4%) Neutropenia (100%) Anemia (100%) Leukopenia (100%) Thrombocytopenia (100%) Lymphopenia (100%)	(51)
			NCT05066646; FUMANBA-1; ChiCTR2000033946 (Phase Ib/II)	CRS (94.9%) Neutropenia (88.6%) Anemia (64.6%) Leukopenia (87.3%) Thrombocytopenia (75.9%) Lymphopenia (74.7%) hypogammaglobulinemia (88.6%)	(50)
Zevorcabtagene Autoleucel	CAFA therapeutics and HK inno.N	Phase II	LUMMICAR study 1; NCT03975907; CT053-MM-01 (Phase I/II)	CRS (90.2%) Neutropenia (93.1%) Leukopenia (91.2%) Thrombocytopenia (84.3%) Lymphopenia (84.3%)	(49)
			LUMMICAR study 2; NCT03915184; CT053-MM-02 (Phase Ib/II)	CRS(59%) Neurologic toxic effect (17.6%)	
Anito-cel	Gilead Sciences	Phase III	NCT04155749 (Phase I)	Neutropenia (81.6%) Anemia (57.9%) Thrombocytopenia (42.1%) Lymphopenia (39.5%)	
Bb21217	Bluebird Bio	R&D terminated	NCT03274219 (Phase I)	CRS (75%) Neurologic toxic effect (15%)	(52)
Orva-cel (JCARH125)	Juno Therapeutics, a Subsidiary of Celgene	Phase II	NCT03430011 (Phase I/II)	CRS (98%) Neutropenia (55%) Anemia (21%) Thrombocytopenia (44%)	(53)
P-BCMA-101	Poseida Therapeutics, Inc	Phase I/II	Phase I/II; NCT03288493; PRIME study	CRS (25%) Neurologic toxic effect (7%) Neutropenia (74%) Anemia (35%) Thrombocytopenia (30%)	(48)
ALL0-715	Allogene Therapeutics	Phase I	NCT04093596 (Phase I)	CRS (55.8%) Neurologic toxic effect (14%) Neutropenia (69.8%) Anemia (55.8%) Leukopenia (11.6%) Thrombocytopenia (51.2%) Lymphopenia (32.6%)	(54)

A summary of the major adverse effects of BCMA-targeted CAR T cells, which are on the market or in different research stages. Major adverse effects, which occurrence rate is more than 10%.

Preclinical safety and efficacy studies are essential to translating T-cell therapy into clinical application (44). In the current study, we evaluated the delayed toxicity of BCMA CAR T-cell injection in a B-NDG immune deficiency mouse model of MM. Our data suggest that BCMA CAR T cells do not disrupt host homeostasis in a range of dosages. The results of tissue distribution studies showed that CAR genes were distributed in different tissues, with the highest levels in the kidney and bone marrow, consistent with the metastatic pathway of MM cancer cells, demonstrating the specific targeting of BCMA CAR T cells to MM. At the same time, histological assessment showed that T-cell accumulation does not lead to significant pathological changes in multiple organs, indicating that BCMA CAR T cells do not have non-specific off-target activity or cross-reactivity. These data provide information on the safety of BCMA CAR T-cell injection in the treatment of MM, especially the CRS risk suggested lower than the report, and our findings support the clinical development of BCMA CAR-T for the treatment of patients with relapsed/refractory MM. Our study may provide an important part of the information for the filing of an investigational new drug for BCMA CAR T-cell injection. In this study, we used a human cancer xenograft mice model, results elucidated the efficacy and safety of CAR T-cell therapy in solving blood cancer, especially before the first in human study. However, there are also some limitations in the study, the xenograft models do not completely reproduce the complex human immune environment, making it difficult to predict severe immune-related toxicities, such as inflammation-associated CRS and neurotoxicity, which frequently occur in clinical trials. Various kinds of cytokines participate in the immunotherapy response, granulocyte-macrophage colony-stimulating factor, interleukins, other factors, and so forth. The models cannot predict all the interactions between human immune cells and CAR T cells, which may assess the toxic effects of CAR-T therapy. It is suggested that it is necessary to use a comprehensive assessment system including *in-vivo*, *in-vitro*, and *ex-vivo* methods to predict the safety risks, such as humanized mice, mini-organs, and organ-on-a-chip (50). The development and modification of preclinical models are essential to bridge the gap between early testing and human clinical trials and to more safely advance the clinical application of engineered T-cell therapies. In future studies, we may try to find more suitable models to study additional adverse effect risks of CAR T cells.

## Conclusions

In the present study, the results of the MM mouse toxicity tests demonstrated that BCMA CAR T cells could well alleviate the clinical symptoms, maintain body weight and prolong the survival of MM mice. We conclude that the BCMA CAR T-cell injection in this study has good anticancer activity and safety. The current data can provide support for future clinical studies and is suitable for further development and application of the BCMA CAR T cells.

## Data Availability

The raw data supporting the conclusions of this article will be made available by the authors, without undue reservation.
